# Supplementary material for: Locating Temporal Functional Dynamics of Visual Short-Term Memory Binding using Graph Modular Dirichlet Energy
Source: Sci Rep. 2017 Feb 10;7:42013. doi: 10.1038/srep42013 (PMC5301217; doi:10.1038/srep42013)
Supplement: Supplementary Material [file srep42013-s1.pdf]

Locating Temporal Functional Dynamics of Visual Short-Term Memory Binding using Graph Modular Dirichlet Energy: Supplementary Material

Keith Smith[1,2,*], Benjamin Ricaud[3], Nauman Shahid[3], Stephen Rhodes[4], John M. Starr[2], Augustin Ibáñez[5,6,7,8,9], Mario A. Parra[2,4,7,10], Javier Escudero[1,†] & Pierre Vandergheynst[3,†]

[1]Institute for Digital Communications, University of Edinburgh, West Mains Rd, Edinburgh, EH9 3FB, UK
[2]Alzheimer Scotland Dementia Research Centre, University of Edinburgh, 7 George Square, Edinburgh, EH8 9JZ, UK
[3]Signal Processing Laboratory 2, École Polytechnique Fédérale de Lausanne, 1015 Lausanne, Switzerland
[4]Human Cognitive Neuroscience and Centre for Cognitive Ageing and Cognitive Epidemiology, Department of Psychology, University of Edinburgh, EH8 9JZ, UK
[5]Institute of Translational and Cognitive Neuroscience (INCyT), INECO Foundation, Favaloro University, Buenos Aires, Argentina
[6]National Scientific and Technical Research Council (CONICET), Buenos Aires, Argentina
[7]Universidad Autónoma del Caribe, Barranquilla, Colombia
[8]Center for Social and Cognitive Neuroscience (CSCN), School of Psychology, Universidad Adolfo Ibáñez, Santiago de Chile, Chile
[9]ARC Centre of Excellence in Cognition and its Disorders, Sydney, Australia
[10]Psychology Department, Heriot-Watt University, Edinburgh, EH14 4AS, UK
*k.smith@ed.ac.uk
†These authors share senior authorship

1 Mathematical Formulation of Modular Dirichlet Energy

Let $G = (\mathcal{V}, \mathbf{f}, \mathcal{E}, \mathbf{W})$ be the mathematical representation of an undirected, labeled graph where $\mathcal{V} = \{1, ..., n\}$ is the vertex set of the graph; $\mathbf{f} = \{f_1, f_2, \ldots, f_n\}$ is the graph signal, or vertex amplitudes, indexed by $\mathcal{V}$; $\mathcal{E} = \{(i, j) \text{ s.t } i \text{ is adjacent to } j \text{ for } i, j \in \mathcal{V}\}$ is the edge set with $|\mathcal{E}| = 2m$ and

$$\mathbf{W} = \begin{cases} w_{ij} & (i, j) \in \mathcal{E} \\ 0 & \text{otherwise} \end{cases}$$

is the weighted adjacency matrix of edge weights indexed by $\mathcal{E}$, where the magnitude of w_{ij} is a measure of strength of relationship between nodes i and j. Then we call $(\mathcal{V}, \mathbf{f})$ the vertex space of the graph and $(\mathcal{E}, \mathbf{W})$ the edge space of the graph so that G is a dual space composed of the vertex and edge spaces where every element of the edge set in the edge space is an ordered pair of elements from the vertex set in the vertex space.

An illustration of a graph with a graph signal is shown in Fig. 1. The blue circles represent the nodes of the network and the lines connecting them represent the edges. The graph signal is composed of the node amplitudes which are represented in Fig. 1 by the orange and blue vertical arrows-positive and negative values, respectively.

Modularity

We define a subgraph, $\mathcal{S}_x$, of G as $\mathcal{S}_x = (\mathcal{V}_x, \mathbf{f}_x, \mathcal{E}_x, \mathbf{W}_x)$ such that $\mathcal{V}_x \subset \mathcal{V}$ and $\mathcal{E}_x \subset \mathcal{E}$ where elements of $\mathcal{E}_x$ are ordered pairs of elements in $\mathcal{V}_x$, $\mathbf{f}_x$ is the graph signal indexed by $\mathcal{V}_x$ and $\mathbf{W}_x$ is the weighted adjacency matrix indexed by $\mathcal{E}_x$. Further we define a full subgraph, G_x, of G as a subgraph such that $\mathcal{E}_x = \{(i, j) \in \mathcal{E} \text{ s.t. } i, j \in \mathcal{V}_x\}$. That is, all of the edges that exist between vertices of the graph which lie in the subset, $\mathcal{V}_x$, are exactly those that exist in the full subgraph, G_x.

We now define a module, for some vertex subset, $\mathcal{V}_x$, as the subgraph $\mathcal{G}_x = (\mathcal{V}, \mathbf{f}, \mathcal{H}_x, \mathbf{W}_x)$ of G, where $\mathcal{H}_x = \{(i, j) \in \mathcal{E} \text{ s.t. } i \in \mathcal{V}_x \;\&\; j \in \mathcal{V}\} \subset \mathcal{E}$. This contains the full subgraph of $\mathcal{V}_x$, but also all of the edges for which one of its adjacent vertices is in $\mathcal{V}_x$ but the other exists in $\mathcal{V} \setminus \mathcal{V}_x$.

Now, modularity is concerned with disjoint partitions of a graph by the vertex set. Particularly, from the above definitions, we can decompose a graph into disjoint modules corresponding to a disjoint decomposition of

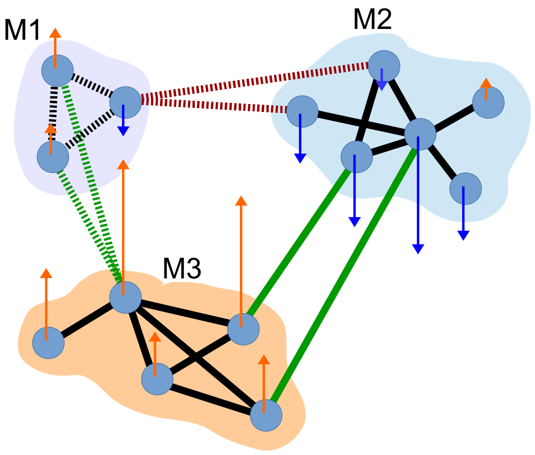

Figure 1: Illustration of a graph with a graph signal, indicated by the orange (positive) and blue (negative) lines at the nodes, decomposed into modules M1, M2 and M3. The dashed lines indicate all the edges associated with module M1.

the vertex set $\mathcal{V} = \bigsqcup_{x=1}^{M} \mathcal{V}_x$ such that

$$G = \bigcup_{x=1}^{M} \mathcal{G}_x = \bigcup_{x=1}^{M} (\mathcal{V}_x, \mathbf{f}_x, \mathcal{H}_x, \mathbf{W}_x).$$

In fact, $\{\mathcal{H}_x\}_{x=1}^{M}$ are mutually disjoint subsets because (i,j) and (j,i), although referring to the same undirected edge in the graph, are separate elements of the edge set $\mathcal{E}$ and appear in separate modules given that i and j are in different subsets of $\mathcal{V}$. Given this property it is useful now to define a set operation we name the edge intersection, $\wedge$, such that

$$\mathcal{H}_x \wedge \mathcal{H}_y = \{(i,j) \in \mathcal{E} \text{ s.t. } i \in \mathcal{V}_x \ \& \ j \in \mathcal{V}_y \text{ or } j \in \mathcal{V}_x \ \& \ i \in \mathcal{V}_y\},$$

$$\mathbf{W}_x \wedge \mathbf{W}_y = \begin{cases} w_{ij} & \text{if } (i,j) \in \mathcal{E} \text{ and either } i \in \mathcal{V}_x \ \& \ j \in \mathcal{V}_y \\ & \text{or } j \in \mathcal{V}_x \ \& \ i \in \mathcal{V}_y \\ 0 & \text{otherwise} \end{cases}$$

and, for two subgraphs, $\mathcal{S}_x$ and $\mathcal{S}_y$, of G,

$$\mathcal{S}_x \wedge \mathcal{S}_y = (\mathcal{V}_x \cup \mathcal{V}_y, \mathbf{f}_x \cup \mathbf{f}_y, \mathcal{H}_x \wedge \mathcal{H}_y, \mathbf{W}_x \wedge \mathbf{W}_y).$$

Modular Dirichlet Energy

The Dirichlet energy of the graph G is defined as

$$E(G) = \sum_{i,j=1}^{n} w_{ij}(f_i - f_j)^2, \tag{1}$$

which is an inverse measure of the smoothness of the graph signal $\mathbf{f}$ over G [1]. Particularly, the node gradient at node i is an important measure of the smoothness of the graph signal at node i and is defined as

$$E(i) = \sum_{j} w_{ij}(f_i - f_j)^2. \tag{2}$$

Note that the elements in the sum of (1) have a one to one mapping to the edge set, $\mathcal{E}$, of G. It follows that there is a natural decomposition of the Dirichlet energy in (1), corresponding to any disjoint composition of the underlying graph into modules, such that

$$E(G) = \sum_{x=1}^{M} \sum_{i \in \mathcal{V}_x} \sum_{j \in \mathcal{V}} w_{ij}(f_i - f_j)^2,$$

and we define the Modular Dirichlet Energy (MDE) of $\mathcal{G}_x$ to be

$$\begin{aligned} MDE(\mathcal{G}_x) &= \sum_{i \in \mathcal{V}_x} \sum_{j \in \mathcal{V}} w_{ij}(f_i - f_j)^2. & (3) \\ &= \sum_{i,j \in \mathcal{V}_x} w_{ij}(f_i - f_j)^2 + \sum_{p \in \mathcal{V}_x} \sum_{q \in \mathcal{V} \setminus \mathcal{V}_x} w_{pq}(f_p - f_q)^2, & (4) \end{aligned}$$

so that the sum of MDEs equals the total Dirichlet energy in the graph (3), satisfying energy preservation, and the MDE accounts both for the Dirichlet energy within the module and the Dirichlet energy of the module interacting with the rest of the graph (4), which directly corresponds to modules in networks. The dashed lines in Fig. 1 represent all the edges, and corresponding Dirichlet energy components, of the module M1. Further, we can define the within module Dirichlet energy and between module Dirichlet energy (BMDE) as

$$E(G_x) = \sum_{i,j \in \mathcal{V}_x} w_{ij}(f_i - f_j)^2, \tag{5}$$

and, for two disjoint modules $\mathcal{G}_x$ and $\mathcal{G}_y$,

$$BMDE(\mathcal{G}_x, \mathcal{G}_y) = \sum_{i \in \mathcal{V}_x} \sum_{j \in \mathcal{V}_y} w_{ij}(f_i - f_j)^2, \tag{6}$$

respectively. In Fig. 1, the black lines represent within module edges and energy components of each module and the dashed brown lines indicate the between module edges and energy components of modules M1 and M2. Now,

$$MDE(\mathcal{G}_x) = E(G_x) + \sum_{y \neq x} BMDE(\mathcal{G}_x \wedge \mathcal{G}_y), \tag{7}$$

and

$$E(G) = \sum_{x=1}^{M} \left(E(G_x) + \sum_{y \neq x} BMDE(\mathcal{G}_x \cap \mathcal{G}_y) \right). \tag{8}$$

That is, the MDE at module $\mathcal{G}_x$ of G is the within module Dirichlet energy, or Dirichlet energy of the subgraph, G_x, combined with the sum of the between module Dirichlet energies from $\mathcal{G}_x$ to all other modules in the graph (7), which we call the Modular Interaction Dirichlet Energy (MIDE), i.e.

$$MIDE(\mathcal{G}_x) = \sum_{y \neq x} BMDE(\mathcal{G}_x \cap \mathcal{G}_y). \tag{9}$$

The green lines of Fig. 1 represent the interaction edges and energy components of module M2. Further, the total Dirichlet energy of G is the sum of all within module Dirichlet energies and BMDEs (8). Note that the elements in $(\mathcal{V}_x, \mathcal{H}_x) \wedge (\mathcal{V}_y, \mathcal{H}_y)$ corresponds exactly to those elements in the sum of (6).

For graph signals which also have a temporal dimension, i.e. $\mathbf{F} = [\mathbf{f}^0, \mathbf{f}^1, \ldots, \mathbf{f}^Y]$, an $n \times Y$ matrix of chronologically ordered graph signals $\mathbf{f}^i = \{f_1^i, f_2^i, \ldots, f_n^i\}$, the Dirichlet energy of the signal during time period $[t_0, t]$ is just the sum of the individual Dirichlet energies at each point in time:

$$E(G)|_{[t_0,t]} = \sum_{s=t_0}^{t} \sum_{i,j=1}^{n} w_{ij}(f_i^s - f_j^s)^2.$$

Thanks to linearity, this extends straightforwardly to all definitions above. By looking at short intervals of graph signals, $[t_0, t]$, we can study moments in time of the network behaviour by looking at the graph signal in the short interval acting over the graph defined by connectivity of the whole epoch and thus probe the connectivity information for dynamic behaviour within the epoch on which the graphs are constructed.

Computation of Modular Dirichlet Energy

It is known that for a graph, G, with Laplacian, $\mathbf{L} \in \mathbb{R}^{n\times n}$ and signal $\mathbf{F} \in \mathbb{R}^{n\times Y}$, the Dirichlet energy is $E(G) = 2\text{tr}(\mathbf{F}^T\mathbf{L}\mathbf{F})$ [1], where tr() is the trace function for matrices. Note, the multiplication by 2 comes from the fact that w_{ij} and w_{ji} relate to edges (i,j) and (j,i) which are regarded as separate elements. This then corresponds with the theoretical formulation of a graph, although, in fact, they are always equal in an undirected graph and relate to the same undirected edge. Now, the within module Dirichlet energy of module $\mathcal{G}_x$ is just

$$E(G_x) = 2\text{tr}(\mathbf{F}_x^T\mathbf{L}_x\mathbf{F}_x),$$

where $\mathbf{L}_x$ is the Laplacian of full subgraph G_x and $\mathbf{F}_x$ is the subset of $\mathbf{F}$ corresponding to the vertex subset $\mathcal{V}_x$.

To compute $E(\mathcal{G}_x)$ easily, we wish to express it also in terms of operations on the Laplacian. We can do this straightforwardly using basic set theoretic relationships on the underlying graph because the elements $\{w_{ij}(f_i - f_j)^2\}_{(i,j)\in\mathcal{E}}$ constitute a set with a one to one mapping to the edge set, $\mathcal{E}$. Let $A = E(G_x)$, $B = E(G_{\backslash x})$ and $2C = E(\mathcal{G}_x \wedge \mathcal{G}_y)$, where $G_{\backslash x}$ is the full subgraph whose vertex set is the complement $\mathcal{V}_x$ and we choose $2C$ because (i,j) and (j,i) are treated as separate elements both with corresponding elements in $E(\mathcal{G}_x \wedge \mathcal{G}_y)$. Then we know how to compute A, B and $A + B + 2C = E(G)$ and we find $E(\mathcal{G}_x)$ as

$$\begin{aligned}
MDE(\mathcal{G}_x) &= A + C \\
&= A + \tfrac{1}{2}((A + B + 2C) - A - B) \\
&= \tfrac{1}{2}(A + (A + B + 2C) - B) \\
&= \tfrac{1}{2}(E(G_x) + E(G) - E(G_{\backslash x})) \\
&= \text{tr}(\mathbf{F}_x^T\mathbf{L}_x\mathbf{F}_x + \mathbf{F}^T\mathbf{L}\mathbf{F} - \mathbf{F}_{\backslash x}^T\mathbf{L}_{\backslash x}\mathbf{F}_{\backslash x}),
\end{aligned}$$

Following from this, it is straightforward to see that the MIDE of a module can be expressed as

$$\begin{aligned}
MIDE(\mathcal{G}_x) &= MDE(\mathcal{G}_x) - E(G_x) \qquad (10) \\
&= MDE(\mathcal{G}_x) - 2\text{tr}(\mathbf{F}_x^T\mathbf{L}_x\mathbf{F}_x) \\
&= \text{tr}(\mathbf{F}^T\mathbf{L}\mathbf{F} - \mathbf{F}_x^T\mathbf{L}_x\mathbf{F}_x - \mathbf{F}_{\backslash x}^T\mathbf{L}_{\backslash x}\mathbf{F}_{\backslash x}),
\end{aligned}$$

where (10) comes from (7). Finally, taking $\mathcal{G}_x \cup \mathcal{G}_y = (\mathcal{V}_x \cup \mathcal{V}_y, \mathbf{F}_x \cup \mathbf{F}_y, \mathcal{H}_x \cup \mathcal{H}_y, \mathbf{W}_x \cup \mathbf{W}_y)$ to be the module of the union of modules $\mathcal{G}_x$ and $\mathcal{G}_y$, the between module dirichlet energy of $\mathcal{G}_x$ and $\mathcal{G}_y$ is

$$BMDE(\mathcal{G}_x, \mathcal{G}_y) = \text{tr}((\mathbf{F}_x \cup \mathbf{F}_y)^T\mathbf{L}_{x\cup y}(\mathbf{F}_x \cup \mathbf{F}_y) - \mathbf{F}_x^T\mathbf{L}_x\mathbf{F}_x - \mathbf{F}_y^T\mathbf{L}_y\mathbf{F}_y).$$

2 Frequency Band Analysis

We detail the frequency band analysis of Alpha, Beta and Gamma for the dataset explored in our manuscript. These are the bands seen as important in visual processing [2]. The methodology follows exactly the procedure in the manuscript. The only addition is that we filter the mean signals with an order 70 FIR filter in Alpha (8-13Hz), Beta (13-32Hz) and Gamma (32-40Hz) prior to connectivity analysis. The results are shown in Tables 1 and 2.

Only one result is apparent after fdr in level 1 of Alpha in the Frontal region. None of the 2nd level MDE results survive the fdr procedure for this case. It is notable that Beta, frontal right, shows some sensitivity also indicating that the effect is likely spread between Alpha and Beta frequencies, justifying our initial broadband analysis. We conclude that the activity is best understood under the original broadband analysis as splitting the analysis into frequency bands weakens sensitivity.

3 Module Selection

In this section we present two tables detailing the sensitivity of module selection in this study.

Table 3 provides evidential justification of the scalp regions selected for analysis by providing extended t-test results for other regions- 'Frontal-Polar' indicates the module consisting of electrodes FP1 and FP2; 'T. Left' is F7, FT7, T3 and TP7; 'T.Right' is F8, FT8, T4 and TP8; 'Parieto-Central' is C3, Cz, C4, CP3, CPz and CP4; 'Parietal' is CP3, CPz, CP4, P3, Pz and P4. As is evident, none of these modules picked up any difference in activity between the Shape and Binding tasks in either the left hemifield or right hemifield conditions except the Frontal Polar which shows only a weak result in the right hemifield and with which the MDE analysis finds no meaningful epochs. This clarifies that we did not miss out on important activity happening elsewhere in the EEG signal correlates and also highlights the anatomical specificity of the activity elicited during this memory paradigm.

Table 4 shows the robustness of results found to small adjustments in size of module selection. We consider both smaller sizes where we eliminate one electrode from the module in the analysis, and larger sizes where we add one neighbouring electrode into the module. As is evident, the results are robust to small changes in the module size, showing that the physio-

logical considerations for electrode choice in modules do not substantially influence the results of the study. This stability can be understood as a direct consequence of our approach to use modules, i.e. a weighted combination of electrodes, as opposed to single electrode analysis. Further, the mean over all p-values for the frontal and occipital modules, including the originals in the study, are 0.0060 and 0.0117, respectively, which are both higher than the p-values of the original study (0.0044 and 0.0102, respectively). This highlights that the bulk of the important functional activity is indeed covered by our chosen modules.

4 Extended 2nd Level results

Extended results of for the hypothesis tree including the left hemifield conditions are included in Table 5.

References

[1] Shuman, D., Narang, S.K., Frossard, P., Ortega, A., Vandergheynst, P., The emerging field of signal processing on graphs. *IEEE Signal Processing Magazine*, **30(3)**: 83-98 (2013).

[2] Palva, S., Palva, J.M., New vistas for α-frequency band oscillations, *Trends in Neuroscience*, **30(4):** 150158 (2007)

Table 1: p-values for paired t-tests of modular sum of edge weights in Shape vs. Shape-colour binding conditions in the encoding perion (0-200ms). O = occipital module, F = frontal module, L = left hemifield condition, R = right hemifield condition. Underlined = true discovery

**Band**	**O.L**	**O.R**	**F.L**	**F.R**
Alpha	0.2528	0.0212	0.7002	<u>0.0035</u>
Beta	0.8104	0.1085	0.6919	0.0085
Gamma	0.1253	0.7449	0.5604	0.4503

Table 2: p-values for paired t-tests of Modular Dirichlet Energy (MDE) in Shape vs. Shape-colour binding conditions. Legend as in Table 1.

**Epoch (ms)**	**0-20**	**20-40**	**40-60**	**60-80**	**80-100**
**Alpha F.R**	0.1333	0.4013	0.5775	0.0800	0.0699
**Epoch (ms)**	**100-120**	**120-140**	**140-160**	**160-180**	**180-200**
**Alpha F.R**	0.1002	0.0081	0.0834	0.0651	0.0175

Table 3: p-values for paired t-tests of modular sum of edge weights in Shape vs. Shape-colour binding conditions in both hemifields for the indicated modules.

**Module**	**Left hemifield**	**Right hemifield**
Frontal-Polar	0.0519	0.0488
T. Left	0.3932	0.6468
T. Right	0.6745	0.8300
Parieto-Central	0.6894	0.5036
Parietal	0.9115	0.5735

Table 4: p-values for paired t-tests of modular sum of edge weights in Shape vs. Shape-colour binding conditions in the right hemifield for the smaller and larger modules indicated by the module in the left column ± 'electrode' in the corresponding entry above the p-value.

**Frontal**	-F3	-Fz	-F4	-FC3	-FCz	-FC4
**p-value**	0.0033	0.0055	0.0050	0.0051	0.0039	0.0067
**Frontal**	+FP1	+FP2	+F7	+F8	+FT7	+FT8
**p-value**	0.0036	0.0076	0.0076	0.0069	0.0086	0.0103
**Occipital**	-O1	-Oz	-O2	-PO1	-PO2	-
**p-value**	0.0126	0.0077	0.0190	0.0077	0.0101	-
**Occipital**	+P3	+Pz	+P4	-	-	-
**p-value**	0.0121	0.0136	0.0126	-	-	-

Table 5: p-values for paired t-tests of Modular Dirichlet Energy (MDE) and Between MDE (BMDE) in Shape vs. Shape-colour binding conditions in encoding. R = Right, L = Left, F = Frontal module, O = Occipital module.

**Time (ms)**	**MDE - O.R**	**MDE - F.R**	**BMDE - F.O.R**
**0-20**	0.2036	0.4088	0.0942
**20-40**	0.0909	0.3891	0.0957
**40-60**	0.0432	0.1380	0.1408
**60-80**	0.0718	0.8074	0.1805
**80-100**	0.0254	0.1918	0.0412
**100-120**	0.0038	0.0465	0.0073
**120-140**	0.0010	0.0851	0.0028
**140-160**	0.0278	0.0070	0.0120
**160-180**	0.0919	0.0059	0.0167
**180-200**	0.6661	0.5464	0.9644

**Time (ms)**	**MDE - O.L**	**MDE - F.L**	**BMDE - F.O.L**
**0-20**	0.6311	0.9355	0.4809
**20-40**	0.9765	0.5652	0.7915
**40-60**	0.1608	0.5770	0.1893
**60-80**	0.2141	0.1872	0.2413
**80-100**	0.1601	0.2711	0.1753
**100-120**	0.4023	0.1890	0.1154
**120-140**	0.8934	0.5647	0.4847
**140-160**	0.8667	0.2155	0.7921
**160-180**	0.9890	0.3413	0.9423
**180-200**	0.7782	0.4726	0.6740
